# Morphological ant mimics: constrained to imperfection?

**DOI:** 10.1098/rsbl.2023.0330

**Published:** 2024-02-14

**Authors:** Donald James McLean, Gerasimos Cassis, Marie E. Herberstein

**Affiliations:** ^1^ School of Natural Sciences, Macquarie University, North Ryde, New South Wales 2109, Australia; ^2^ Evolution and Ecology Research Centre, University of New South Wales, Sydney, New South Wales 2052, Australia

**Keywords:** evolutionary constraint, adaptation, imperfect mimicry, myrmecomorphy

## Abstract

Adaptive evolution relies on both heritable variation and selection. Variation is the raw material upon which selection acts, so any mechanism that limits or prevents the generation of heritable variation reduces the power of selection to lead to adaptation. Such limitations are termed evolutionary constraints. While it is widely accepted that constraints play an important role in shaping evolutionary outcomes, their relative importance, as opposed to adaptation, in determining evolutionary outcomes remains a subject of debate. Evolutionary constraints are often evoked as the reason behind the persistence of inaccurate mimicry. Here, we compared the variation and accuracy of body-shape mimicry in ant-mimicking spiders with that of ant-mimicking insects, predicting greater constraints, and hence inaccuracy, in spiders mimicking ants, due to their evolutionary distance from the ant model. We found high inter-species variation in mimetic accuracy, but dorsally, no overall difference in mimetic accuracy between spider and insect mimics, which is inconsistent with a constraint causing inaccurate mimicry. Our study provides empirical evidence suggesting that imperfect mimicry in spiders and insects is predominantly shaped by adaptive processes rather than constraints or chance. Our findings contribute to our understanding of the mechanisms underlying evolutionary diversity and the processes that shape phenotypic outcomes.

## 1. Introduction

Adaptive evolution occurs when selection acts on heritable variation, while an evolutionary constraint is any restriction or bias on the generation of heritable variation [1]. Constraints have long been recognized as affecting evolution [2], however opinions differ as to the relative importance of constraints and historical contingency [3], as opposed to adaptation [4], in determining evolutionary outcomes. Contingency, in this context, refers to both chance and the sensitivity of evolutionary outcomes to small differences in the history of lineages [5]. Within evolutionary literature, the term ‘constraint’ is applied broadly and imprecisely [1,6]. We use the term here to mean a mechanism that limits or biases heritable variation but exclude mechanisms that affect the fitness function, such as selective trade-offs, which are sometimes labelled as constraints [e.g. 7]. We do this to help establish the relative contributions of constraint and adaption on evolutionary diversity.

A constraint prevents a trait from evolving to attain its selective optimum [1,8], so non-optimal traits can be used to identify constraints [8]. Our study uses the exceptional opportunity afforded by imperfect Batesian mimicry. Batesian mimics evolve a phenotypic similarity to their models as greater similarity affords a selective advantage when predators are deceived into rejecting these mimics as potential prey. The theoretical ‘optimal’ Batesian mimetic phenotype is indistinguishable from its model, yet humans can distinguish many, if not most, mimics from their models [9]. One explanation is that constraints prevent imperfect mimics from attaining their ideal phenotype. Of course, there are many possible explanations for imperfect mimicry beyond constraint, many of which are adaptive, meaning that an *imperfect* phenotype carries greater selective benefits than a *perfect* one [9].

**Figure 1.**
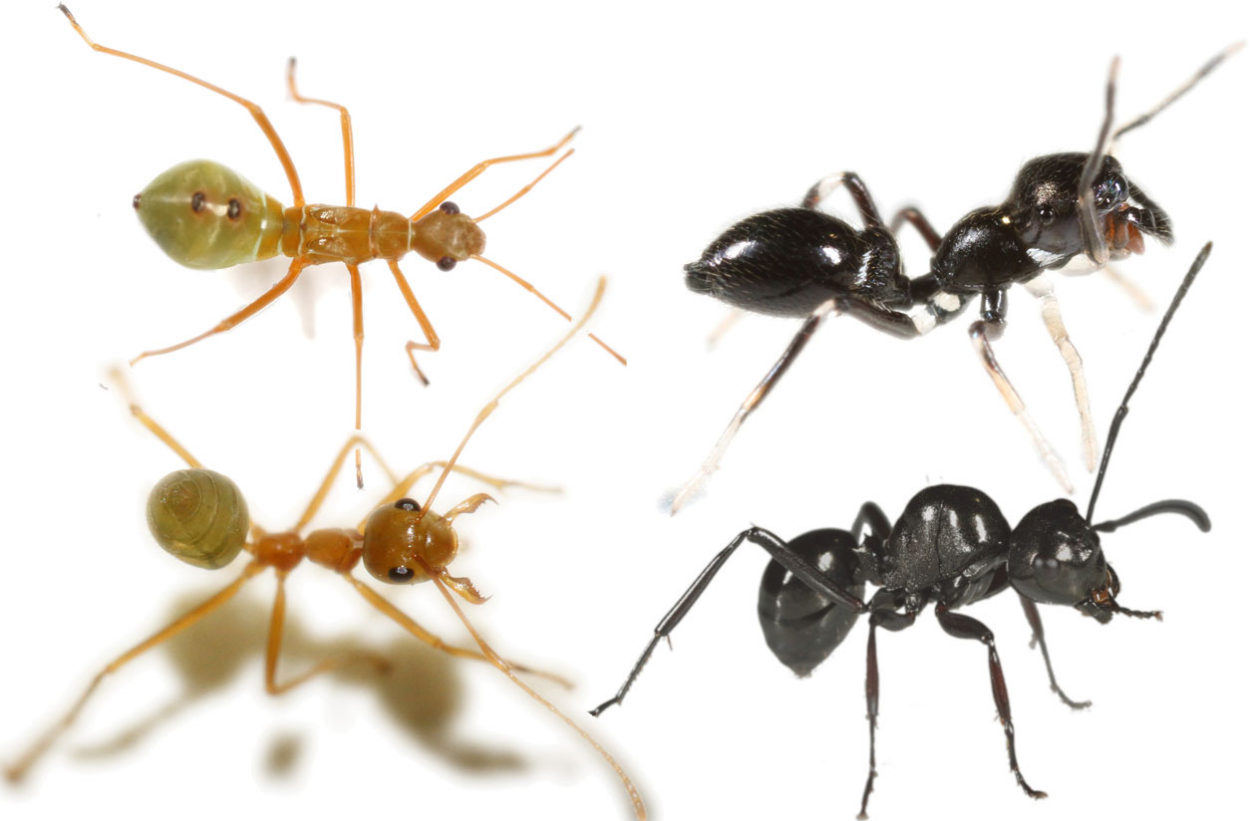
Some ant mimics and putative models. An accurate ant mimicking insect (immature *Alydid* sp., top left) and presumed model (*Oecophylla smaragdina*, bottom left). An accurate ant-mimicking spider (*Myrmarachne macleayana*, top right) and putative ant model (bottom right, *Polyrhachis robsoni*). Photographs, Jim McLean.

Here, we use a comparative approach to look for evidence of constraint [8] in ant mimics (figure 1). Ants are conspicuous, well-armed, often aggressive, distasteful, and attack in concert, all of which makes them unsuitable prey and suitable models for Batesian mimicry [10,11]. Accordingly, ant mimicry is common, comprising as much as 2% of some arthropod taxa [11]. General morphology, and body shape in particular, are considered important aspects of visual ant mimicry [11], so we can look for evidence of constraints, as opposed to adaptive explanations for imperfect mimicry, by comparing the accuracy of body shape ant mimicry in spider and insect species that mimic ants. We hypothesize that some adaptive limitations to accuracy may apply to both groups of taxa, but that constraints will affect spiders more than insects due to their greater evolutionary and morphological distance from ants. The ancestors of spiders and insects diverged at least 500 million years ago [12,13] and morphologically, spiders have two stocky tagma to the three thin tagma of insects.

If spiders are more constrained from attaining greater ant shape mimetic accuracy than insect ant-mimics, we predict that: (1) spiders will have lower mimetic accuracy than insects; and (2) spiders will have less variation in accuracy [14], since constraints prevent variation towards higher accuracy and selection would reduce variation toward lower accuracy (in spiders as in insects).

## 2. Material and methods

We used body shape data that were published previously in a paper that evaluated the use of geometric morphometrics for quantifying accuracy of body-shape mimicry [15]. Our methods are based on the geometric morphometric methods of Kelly *et al.* [15], with modifications. Our methods are summarized here, and for clarity we have included the specimen list as an appendix to this paper (electronic supplementary material, table S1). We collected multiple species of ant-mimicking spiders and insects, ants and non-mimetic arthropods from multiple locations along the east coast of Australia with some preserved specimens borrowed from the Australian Museum. Specimens were identified to species when possible, otherwise to family or genus and morphospecies, using multiple sources [16–19]. Some specimens published in Kelly *et al.* [15] were not identified to this level, so we excluded them from this study. We classified species as visual mimics based on published assessments where possible, although personal judgement was used for several insect mimics. Specimens were photographed using a Canon 7D with an MP-E 65 mm macro lens and an MT-24EX flash. Photographs were taken from both dorsal and lateral aspects, as selective forces and constraints are likely to differ with aspect.

We used geometric morphometric analyses to quantify body shapes. Morphometrics is a quantitative method of addressing shape variation and comparisons [20] that has traditionally involved the use of ‘landmarks’, a set of structurally homologous points on each specimen. Since our specimens lack consistent structural homologies (e.g. spiders lack the petiole or neck of ants), we instead used elliptical Fourier analysis to quantify whole outlines [21]. Photographs were prepared for morphometric analysis by manually converting them (in Adobe Photoshop CS2) to a monochrome image with a solid black body shape, excluding appendages, on a white background. Body outlines were rotated so that the body was horizontal with the head to the left and cropped to leave a small margin. If the body was twisted or bent, we straightened it out so that the body segments were aligned. Any obscured portions of the outlines were manually interpolated. Outline images were resized to 1200 × 800 pixels then converted to monochrome.

To perform the morphometric analysis, outline coordinates were extracted from the image files. Outlines were subsampled to 1600 points, then smoothed to remove noise using five iterations of a sliding average. The resulting shapes were aligned using a Procrustes superimposition, followed by an elliptical Fourier transform, then the Fourier coefficients were normalized to be invariant to body size, rotation and outline starting position. Since our body shapes were already roughly aligned, our normalization method prevented rotation by 180°, which differs slightly from the normalization implemented in the efourier_norm function from the Momocs R package [22]. Our animals have bilateral symmetry, so any asymmetry in the dorsal outlines is an artefact of the animal pose or camera position. Accordingly, we removed asymmetry in the dorsal shapes by setting the Fourier coefficients that represent asymmetry to 0 [23]. The Fourier coefficients for multiple outlines were averaged per specimen, then specimen coefficients averaged to obtain species average shapes. A principal component analysis (PCA) was applied to the coefficients, and we retained the components that represented at least 95% of the variation; this eliminated constant dimensions and reduced the number of data points. The output from this process is a set of shapes represented as *n*-dimensional vectors that identify locations within morphospace. The distance between two locations in morphospace identifies their similarity: similar shapes are close together, while different shapes are further apart.

An index of mimetic accuracy was constructed by calculating the Mahalanobis distance from each shape to the distribution of ant shapes. We normalized the distances by dividing by the distance to the least ant-like shape, then used the inverse as our measure of accuracy. Therefore, our accuracy index (separate for dorsal and ventral aspects) ranged from one, a perfect average ant shape, to zero, the least ant-like.

We computed the bias-corrected and accelerated (BCa) bootstrapped confidence intervals for the differences in group (spider and insect) means and used an F-test to compare the variances in accuracy of spiders and insects, testing whether the ratio of variances is significantly different from 1. Comparative studies across multiple taxa commonly apply phylogenetic correction techniques to correct for phylogenetic non-independence. In our study, many specimens could not be identified to the species level (see methods), meaning that their position in a phylogeny is unknown, ruling out phylogenetic correction. We address the possible implications of not applying phylogenetic correction in the discussion. Calculations and plots were performed in R (version 4.2.2) [24] using the Durga (version 1.1.0) and Momocs (version 1.4.0) packages [22,25].

## 3. Results

**Figure 2.**
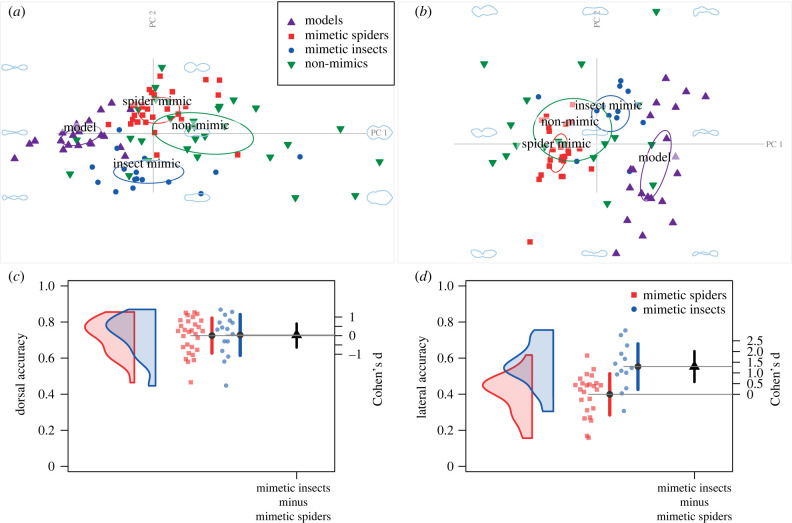
Dorsal (*a*) and lateral (*b*) species' average shapes plotted in morphospace. The *x* and *y* axes are the first two principal components, which account for 71% and 55% of the total variation in the dorsal and lateral data sets, respectively. Ellipses show 95% confidence regions for ant models, spider mimics, insect mimics and non-mimics. The light-coloured shapes graphically indicate the morphospace dimensions of the axes. A single ant has been excluded from the lateral morphospace plot for clarity. (*c*) Dorsal and (*d*) lateral distributions of mimetic accuracy for species’ average shapes, represented as probability density plots (rotated 90°) and truncated to the range of the data. Mimetic accuracy increases along the *y*-axis. Large black dots represent sample means, and vertical error bars show the 95% CI of the means. Large black triangle is the estimate of difference in means, expressed as Cohen's d*.* Error bars show 95% CI.

In total, we used 637 outlines for 248 individuals and 106 species (electronic supplementary material, table S1). The plots of species' shapes in principal component reduced-morphospace revealed clusters for ant models, spider mimics and insect mimics, with lateral body shapes less tightly clustered than dorsal shapes (figure 2*a,b*). The dorsal shapes of mimetic spiders and mimetic insects lie between those for ants and non-mimics, whereas the lateral shapes for mimetic insects lie between the shapes of ants and mimetic spiders.

From a dorsal aspect, mimetic accuracy ranged from 0.47 to 0.85 for spiders and from 0.45 to 0.87 for insects. The mean accuracy of body shape mimicry of spiders was not significantly lower than the mean accuracy of insects (insect–spider mean accuracy Cohen's d = 0.03, 95% CI [−0.67, 0.69]). There was less variance in the dorsal accuracy of spiders, but not significantly so (one-tailed *F*-test, *F*_28, 15_ = 0.74, 95% CI [0, 1.49], *p* = 0.23; figure 2*c*). From a lateral perspective, spider accuracy ranged from 0.16 to 0.61 and insect accuracy from 0.31 to 0.75. On average, spiders were significantly poorer mimics laterally (insect–spider mean accuracy Cohen's d = 1.29, 95% CI [0.37, 1.94]), although the most accurate spider was more accurate than the majority of insect mimics. Laterally, spiders were not significantly less variable in mimetic accuracy than ant mimicking insects (one-tailed F-test, *F*_23, 12_ = 0.82, 95% CI [0, 1.79], *p* = 0.32; figure 2*d*).

## 4. Discussion

Our analysis revealed substantial variation in mimetic quality, from very poor to good, amongst both ant-mimicking spiders and insects, which is consistent with results in mimetic hoverflies [26,27], mimetic snakes [28], and egg mimicry in avian brood parasites [29]. For both groups of mimics (spiders and ants), accuracy is distributed across a continuum, with no clear groupings into good and poor mimics. Several of the poorest mimics (e.g. *Eilica* sp1, *Zodarid4* sp1, *Apricia jovialis* and *Colobathristid1* sp1), scored so poorly that they may not be morphological mimics, although subjectively they appear to match their putative models in colour, which was not considered in this analysis.

The evolutionary constraints hypothesis predicts that constrained mimics will be less accurate and have lower phenotypic variation than non-constrained mimics. Low variation is expected because the constraint prevents greater accuracy from appearing in the population, while selection eliminates variation for lower accuracy. We expected that ant-mimicking spiders would be more likely to experience constraints on body shape mimicry compared with ant-mimicking insects because of their considerable phylogenetic and morphological distance from the model. However, our results do not support this expectation. The most accurate spider mimics are almost as good as the most accurate insect mimics, both dorsally and laterally (figure 2*c,d*). While spiders do have lower average and maximum accuracy than insects from a lateral aspect, there is no reduction in variation. Our results support the contention that evolutionary constraints are unlikely to contribute to imperfect mimicry [30,31], and indeed, that most constraints can be overcome given strong enough selection and adequate time [8].

Phylogenetic correction is unlikely to change our test outcomes or conclusions. Lack of phylogenetic correction may bias effect size estimates or incorrectly reduce confidence intervals. Three of our four statistical tests did not find evidence of an effect, so increasing the width of the confidence intervals would not affect the test results. The fourth test found lower lateral accuracy in spiders. Even if this result were a type I error caused by a lack of phylogenetic correction, this would strengthen, rather than weaken, our general argument against constraints as a mechanism for maintaining imperfect mimicry.

Why are spiders worse mimics than insects from a lateral aspect? If a constraint on lateral accuracy does exist, the limit is imposed at a high level of accuracy since the most accurate spider mimic is more accurate than the majority of insects. The long tail of inaccurate spider mimics in the distribution suggests that selection for accurate lateral mimicry is not strong. If there is reduced selection for mimicry from a lateral aspect on spider and insect mimics, then intrinsic differences in body shape between spiders and insects (two stocky body parts versus three relatively thinner ones) might explain why spider's body shapes are less ant-like. Alternatively, insects and spiders may suffer predation from different predators, leading to different strengths of selection on each. With major visual predators of spiders being birds and wasps [32], both of which are likely to detect and identify prey from above while flying, spiders may be subject to lower selection for lateral mimicry.

Why are so few mimics good, let alone, perfect? The wide range in accuracy of our ant-mimics, both spiders and insects, suggests that selection for accuracy is relaxed, since selection is predicted to reduce phenotypic variation. There are multiple imperfect mimicry hypotheses that predict a relaxed selection adaptive landscape for mimics [9]. For example, predators may err on the side of caution and trade-off the risk of losing prey (by mistakenly rejecting a poor mimic) against the cost of mistakenly attacking a model, or predators may not have enough information to discriminate between poor mimics and models [33].

A vexed question in evolutionary biology is to identify the underlying mechanisms that are responsible for the diversity of forms we see around us. Is adaptation a sufficient explanation, or is it necessary to invoke contingency and constraint [3]? Gould [3] approached this question by asking what would happen if we could replay life's tape. His expectation was that every replay would ‘lead evolution down a pathway radically different from the road actually taken’. Batesian mimicry can be seen as a natural experiment in replaying life's tape, with many separate lineages converging on mimicking well-defended prey, such as ants, despite approaching from different historical backgrounds [11], so replaying life's tape in these cases has not lead to radically different pathways. Imperfect mimics seem to be examples of evolutionary outcomes that result from adaptation rather than constraint or contingency. Constraint in particular is a confused concept, difficult to demonstrate or test for directly. Our study, plus others, question the extent to which the concept adds to our understanding of the evolution and maintenance of diversity.

## Data Availability

No new data were collected for this study. Data used were a subset of the data published by a previous study and are available at https://zenodo.org/badge/latestdoi/236611664 [15]. The folder ‘GeometricMorphometrics/data’ was used in this study. Source code created for this article (including a README.txt file) is available as electronic supplementary material. Supplementary material is available online [34].
